# Supplementary material for: Multifocal imaging for precise, label-free tracking of fast biological processes in 3D
Source: Nat Commun. 2021 Jul 28;12:4574. doi: 10.1038/s41467-021-24768-4 (PMC8319204; doi:10.1038/s41467-021-24768-4)
Supplement: Supplementary file 1 — Description of Additional Supplementary Files [file 41467_2021_24768_MOESM1_ESM.pdf]

Title: Supplementary Movie 1

Description: Multifocal imaging combined with an EDOF algorithm to visualize grooming D. *melanogaster* (1x magnification, play speed is real time).

Title: Supplementary Movie 2

Description: Multifocal imaging combined with an EDOF algorithm to visualize a foraging *Hydra vulgaris* (4x magnification).

Title: Supplementary Movie 3

Description: Multifocal imaging combined with an EDOF algorithm to visualize a crawling *Amoeba proteus* (10x magnification).

Title: Supplementary Movie 4

Description: Multifocal imaging combined with an EDOF algorithm to visualize a swimming human sperm cell (32x magnification).

Title: Supplementary Movie 5

Description: Top: 3D visualization of the four planes acquired by MFI and the flagellum reconstructed using SpermQ-MF and the calibrated relationship between flagellar width, position on the flagellum, and z-distance to the respective plane (Fig. 3a). Flagella indicated in blue. Positions of sperm heads indicated as yellow spheres. Bottom: Individual plane image acquired by MFI and the flagellum reconstructed in 2D (blue).

Title: Supplementary Movie 6

Description: Flagellar 3D reconstruction of a free-swimming human sperm cell over time based on MFI and SpermQ-MF. The beat plane (gray) was defined by the eigenvectors of the flagellum.

Title: Supplementary Movie 7

Description: Tracking of latex beads flowing around a human sperm cell that was tethered to the cover glass at the head (each trajectory is depicted by a different color). On bottom: selected plane image from the multifocal recording.
