# Supplementary material for: Multifocal imaging for precise, label-free tracking of fast biological processes in 3D
Source: Nat Commun. 2021 Jul 28;12:4574. doi: 10.1038/s41467-021-24768-4 (PMC8319204; doi:10.1038/s41467-021-24768-4)
Supplement: Supplementary file 10 — Reporting Summary [file 41467_2021_24768_MOESM10_ESM.pdf]

nature research

Corresponding author(s): Jan Niklas Hansen (jan.hansen@uni-bonn.de)
Luis Alvarez (luis.alvarez@caesar.de)

Last updated by author(s): Jul 3, 2021

Reporting Summary

Nature Research wishes to improve the reproducibility of the work that we publish. This form provides structure for consistency and transparency in reporting. For further information on Nature Research policies, see our Editorial Policies and the Editorial Policy Checklist.

Statistics

For all statistical analyses, confirm that the following items are present in the figure legend, table legend, main text, or Methods section.

n/a	Confirmed	
[ ]	[x]	The exact sample size (n) for each experimental group/condition, given as a discrete number and unit of measurement
[ ]	[x]	A statement on whether measurements were taken from distinct samples or whether the same sample was measured repeatedly
[x]	[ ]	The statistical test(s) used AND whether they are one- or two-sided<br>*Only common tests should be described solely by name; describe more complex techniques in the Methods section.*
[x]	[ ]	A description of all covariates tested
[x]	[ ]	A description of any assumptions or corrections, such as tests of normality and adjustment for multiple comparisons
[ ]	[x]	A full description of the statistical parameters including central tendency (e.g. means) or other basic estimates (e.g. regression coefficient) AND variation (e.g. standard deviation) or associated estimates of uncertainty (e.g. confidence intervals)
[x]	[ ]	For null hypothesis testing, the test statistic (e.g. F, t, r) with confidence intervals, effect sizes, degrees of freedom and P value noted<br>*Give P values as exact values whenever suitable.*
[x]	[ ]	For Bayesian analysis, information on the choice of priors and Markov chain Monte Carlo settings
[x]	[ ]	For hierarchical and complex designs, identification of the appropriate level for tests and full reporting of outcomes
[x]	[ ]	Estimates of effect sizes (e.g. Cohen's d, Pearson's r), indicating how they were calculated

Our web collection on statistics for biologists contains articles on many of the points above.

Software and code

Policy information about availability of computer code

Data collection	Images were acquired using the software pco.camware version 3.09.
Data analysis	Image processing and analysis were performed in ImageJ (v1.52i, National Institutes of Health), MATLAB 2018b (Mathworks), Rstudio (version 1.2.5019), and R (version 3.6.1). Plots and figures were generated using GraphPad Prism (Version 6.07, GraphPad Software, Inc., La Jolla, CA, USA), OriginPro (Version 9.0.0G, OriginLab Corporation, Northampton, USA), MATLAB 2018b (Mathworks), Adobe Illustrator CS5 (Adobe Systems, Inc., v15.0.0, San Jose, CA, USA), and Affinity Designer (Version 1.9.3, Serif (Europe) Ltd.). The Java-based software was developed and compiled using Eclipse Mars.2 (Release 4.5.2, IDE for Java Developers, Eclipse Foundation, Inc.) and using parts of the source code of SpermQ (v0.1.7). All developed software and the respective source code is available at https://github.com/hansenjn/MultifocalImaging-AnalysisToolbox.

For manuscripts utilizing custom algorithms or software that are central to the research but not yet described in published literature, software must be made available to editors and reviewers. We strongly encourage code deposition in a community repository (e.g. GitHub). See the Nature Research guidelines for submitting code & software for further information.

Data

Policy information about availability of data

All manuscripts must include a data availability statement. This statement should provide the following information, where applicable:

- Accession codes, unique identifiers, or web links for publicly available datasets
- A list of figures that have associated raw data
- A description of any restrictions on data availability

All data supporting the findings of this study are contained in the text, the Figures, and the Supplementary Information. Source data are provided as a supplementary file. Exemplary high-speed video recordings to run the analysis routines, extracted data, and analysis supporting this study have been deposited on

GitHub under the accession code https://github.com/hansenjn/MultifocalImaging-AnalysisToolbox. The GitHub repository has been archived on Zenodo under the digital object identifier (DOI) 10.5281/zenodo.5052538. The raw bead tracking data used to generate Fig. 6 were deposited on figshare.com under the DOI 10.6084/m9.figshare.14587509.v1.

Field-specific reporting

Please select the one below that is the best fit for your research. If you are not sure, read the appropriate sections before making your selection.

- [x] Life sciences
- [ ] Behavioural & social sciences
- [ ] Ecological, evolutionary & environmental sciences

For a reference copy of the document with all sections, see nature.com/documents/nr-reporting-summary-flat.pdf

Life sciences study design

All studies must disclose on these points even when the disclosure is negative.

Sample size	Sample size calculations were not performed. Each experiment was replicated independently at least three times. Of note, this study represents a proof of concept - the concepts were verified by application of the method to different size ranges and species, see for example Fig. 1 (Extended Depth Of Field method). The precision of bead tracking was calculated based on theoretical estimations, measurements of non-moving beads and from studying Brownian motion - all methods revealed similar results. The method to track flagellar in 3D was demonstrated for two different species. The flow field obtained matched theoretical predictions. Because results were highly consistent across multiple methods / species / size ranges / replicates, we consider the sample size to be sufficient for a proof of principle of the method.
Data exclusions	No data were excluded.
Replication	All attempts of replication succeeded. Fig. 1: representative images selected from three independent recordings. Fig. 2: this experiment was performed three times with similar results. Fig. 2a-g: based on twelve beads from four independent recordings. Fig. 2h-j: based on 81 beads from four independent recordings. Fig. 3: based on 15 sperm from five different donors. Fig. 4, 5, Supplementary Fig. 5-10: based on seven human sperm from two different donors and 10 sea urchin sperm from the same animal. Fig. 6, Supplementary Fig. 11: exemplary case randomly selected from 16 recordings of sperm from three different donors. Supplementary Fig. 1: six independent experiments. Supplementary Fig. 3: example case randomly selected from the recordings shown in Fig. 4, 5. Supplementary Fig. 4a: based on five different sperm. Supplementary Fig. 4b: exemplary case from five different sperm. Supplementary Fig. 4c: exemplary case from three different sperm. Supplementary Fig. 4d: based on three different sperm.
Randomization	Samples were randomly acquired. A paradigm for allocation of samples was not applicable to this study because it shows a proof of concept. No datasets are contained in which multiple experimental groups are compared. All the analysis was done automatically.
Blinding	Blinding was not applicable to this study because it shows a proof of concept and no datasets are contained in which multiple experimental groups are compared.

Reporting for specific materials, systems and methods

We require information from authors about some types of materials, experimental systems and methods used in many studies. Here, indicate whether each material, system or method listed is relevant to your study. If you are not sure if a list item applies to your research, read the appropriate section before selecting a response.

Materials & experimental systems

n/a	Involved in the study
x	Antibodies
x	Eukaryotic cell lines
x	Palaeontology and archaeology
	x Animals and other organisms
	x Human research participants
x	Clinical data
x	Dual use research of concern

Methods

n/a	Involved in the study
x	ChIP-seq
x	Flow cytometry
x	MRI-based neuroimaging

Animals and other organisms

Policy information about studies involving animals; ARRIVE guidelines recommended for reporting animal research

Laboratory animals	Amoeba proteus and Hydra vulgaris were purchased freshly before the experiments (Lebendkulturen Helbig, Prien am Chiemsee, Germany). The samples were used as delivered by the company, were not sexed for experiments and were used within a week. Drosophila melanogaster were adults (> 2 days old); Fig. 1 shows a male.
Wild animals	Adult (> 2 years old) male sea urchins from the species Arbacia punctulata were provided by the Marine Resource Center (MRC) at the Marine Biological Laboratory (MBL) in Woods Hole. Animals were transported in sea water to the laboratory on campus. After

	spawning, animals were disposed by the MRC facility of the MBL.
Field-collected samples	The study did not involve animals collected from the field.
Ethics oversight	No ethical approval was required.

Note that full information on the approval of the study protocol must also be provided in the manuscript.

Human research participants

Policy information about studies involving human research participants

Population characteristics	The donors represent a sample of normozoospermic men of the general population (age 20-45).
Recruitment	Human semen samples were donated by adult men with their prior written consent. Only normozoospermic donors were included. There is no self-selecting bias. The identity of the donors was not disclosed to the experimenters.
Ethics oversight	Approval of the ethics committee of the University of Bonn (042/17).

Note that full information on the approval of the study protocol must also be provided in the manuscript.
